# *Pichia kudriavzevii* (*Candida krusei*): A systematic review to inform the World Health Organisation priority list of fungal pathogens

**DOI:** 10.1093/mmy/myad132

**Published:** 2024-06-27

**Authors:** Thi Anh Nguyen, Hannah Yejin Kim, Sophie Stocker, Sarah Kidd, Ana Alastruey-Izquierdo, Aiken Dao, Thomas Harrison, Retno Wahyuningsih, Volker Rickerts, John Perfect, David W Denning, Marcio Nucci, Alessandro Cassini, Justin Beardsley, Valeria Gigante, Hatim Sati, C Orla Morrissey, Jan-Willem Alffenaar

**Affiliations:** Faculty of Medicine and Health, School of Pharmacy, The University of Sydney, Sydney, NSW, Australia; Sydney Infectious Diseases Institute, The University of Sydney, Sydney, NSW, Australia; Faculty of Medicine and Health, School of Pharmacy, The University of Sydney, Sydney, NSW, Australia; Sydney Infectious Diseases Institute, The University of Sydney, Sydney, NSW, Australia; Department of Pharmacy, Westmead Hospital, Sydney, NSW, Australia; Faculty of Medicine and Health, School of Pharmacy, The University of Sydney, Sydney, NSW, Australia; Sydney Infectious Diseases Institute, The University of Sydney, Sydney, NSW, Australia; Department of Clinical Pharmacology and Toxicology, St Vincent's Hospital, Sydney, NSW, Australia; National Mycology Reference Centre, Microbiology and Infectious Diseases, SA Pathology, Adelaide, SA, Australia; Mycology Reference Laboratory, National Centre for Microbiology, Instituto de Salud Carlos III, Majadahonda, Madrid, Spain; Sydney Infectious Diseases Institute, The University of Sydney, Sydney, NSW, Australia; Westmead Institute for Medical Research, Sydney, NSW, Australia; Institute of Infection and Immunity, St George's University London, London, UK; MRC Centre for Medical Mycology, University of Exeter, Exeter, UK; Department of Parasitology, Faculty of Medicine, Universitas Kristen Indonesia, Jakarta, Indonesia; Robert Koch Institute, Berlin, Germany; Division of Infectious Diseases and International Health, Duke University School of Medicine, Durham, NC, USA; Manchester Fungal Infection Group (MFIG), Manchester Academic Health Science Centre, The University of Manchester, Manchester, UK; Department of Internal Medicine, Federal University of Rio de Janeiro, Rio de Janeiro, Brazil; Cantonal Doctor Office, Public Health Department, Canton of Vaud, Lausanne, Switzerland; Sydney Infectious Diseases Institute, The University of Sydney, Sydney, NSW, Australia; Westmead Institute for Medical Research, Sydney, NSW, Australia; AMR Division, World Health Organisation, Geneva, Switzerland; AMR Division, World Health Organisation, Geneva, Switzerland; Department of Infectious Diseases, Alfred Health, Melbourne, VIC, Australia; Department of Infectious Diseases, Central Clinical School, Faculty of Medicine, Nursing and Health Sciences, Monash University, Melbourne, VIC, Australia; Faculty of Medicine and Health, School of Pharmacy, The University of Sydney, Sydney, NSW, Australia; Sydney Infectious Diseases Institute, The University of Sydney, Sydney, NSW, Australia; Department of Pharmacy, Westmead Hospital, Sydney, NSW, Australia

**Keywords:** *Pichia kudriavzevii*, *Candida krusei*, mortality, drug resistance, prevention, epidemiology

## Abstract

In response to the growing global threat of fungal infections, in 2020 the World Health Organisation (WHO) established an Expert Group to identify priority fungi and develop the first WHO fungal priority pathogen list (FPPL). The aim of this systematic review was to evaluate the features and global impact of invasive infections caused by *Pichia kudriavzevii* (formerly known as *Candida krusei*). PubMed and Web of Science were used to identify studies published between 1 January 2011 and 18 February 2021 reporting on the criteria of mortality, morbidity (defined as hospitalisation and length of stay), drug resistance, preventability, yearly incidence, and distribution/emergence. Overall, 33 studies were evaluated. Mortality rates of up to 67% in adults were reported. Despite the intrinsic resistance of *P. kudriavzevii* to fluconazole with decreased susceptibility to amphotericin B, resistance (or non-wild-type rate) to other azoles and echinocandins was low, ranging between 0 and 5%. Risk factors for developing *P. kudriavzevii* infections included low birth weight, prior use of antibiotics/antifungals, and an underlying diagnosis of gastrointestinal disease or cancer. The incidence of infections caused by *P. kudriavzevii* is generally low (∼5% of all *Candida*-like blood isolates) and stable over the 10-year timeframe, although additional surveillance data are needed. Strategies targeting the identified risk factors for developing *P. kudriavzevii* infections should be developed and tested for effectiveness and feasibility of implementation. Studies presenting data on epidemiology and susceptibility of *P. kudriavzevii* were scarce, especially in low- and middle-income countries (LMICs). Thus, global surveillance systems are required to monitor the incidence, susceptibility, and morbidity of *P. kudriavzevii* invasive infections to inform diagnosis and treatment. Timely species-level identification and susceptibility testing should be conducted to reduce the high mortality and limit the spread of *P. kudriavzevii* in healthcare facilities.

## Introduction

Fungal pathogens contribute to a high burden of disease and are major threats to global health. Although the burden has not been accurately measured, crude estimates suggest they cause over 1.6 million deaths annually.^1^ People who are immunocompromised due to cancer, chronic lung disease, tuberculosis, HIV, organ transplantation, major abdominal surgery, or are on immunosuppressive drugs are vulnerable to serious fungal infections.^1,2^ Despite the global concern, the allocation of research support to generate robust data from clinical and microbiological studies to, in turn, support the development of effective diagnosis and treatment strategies for fungal infections has been limited to date. Lack of comprehensive surveillance systems also leaves clinicians in an evidence vacuum, relying on sparse or anecdotal information regarding local epidemiology, antimicrobial resistance, and treatment strategies to inform clinical decision-making.

In recognition of the growing global threat of fungal pathogens, in 2020 World Health Organisation (WHO) established an Expert Group to identify priority fungi and develop the first fungal priority pathogen list (FPPL). The FPPL was developed through a wide international consultation process using a survey composed of discrete choice experiments (DCE). Individual fungal pathogens were subsequently ranked based on the results of the DCE, informed by systematic reviews. This global exercise highlighted the urgent need for prioritising research and interventions against invasive fungal infections.

Invasive fungal diseases (IFD) are associated with mortality and morbidity for hospitalised patients and increased healthcare costs.^1,3,4^ Whilst *Candida* species were a common cause of IFD in previous decades,^2^ an increasing incidence of other yeast-like fungi have been reported more recently.^3,4^ Among the non-*Candida* yeasts, *Pichia kudriavzevii*, which was formerly and is still commonly known as *Candida krusei*, is a rare but well-recognised pathogen due to its intrinsic resistance to fluconazole and decreased susceptibility to amphotericin B.^4,5^*Pichia kudriavzevii* is likely to affect immunocompromised patients and is associated with a high mortality rate (49%).^4–6^ Consequently, *P. kudriavzevii* has been selected among the fungi to rank in the FPPL of the WHO.

Despite these major concerns, limited research has been conducted to support the effective diagnosis and treatment of *P. kudriavzevii* infections. Whilst two recent reviews have focused on the basic science aspects of *P. kudriavzevii*,^4,7^ an update of clinically relevant characteristics and global impact of *P. kudriavzevii* invasive infections is required.

We conducted a systematic review to (1) evaluate the features and global impact of invasive infections caused by *P. kudriavzevii*, and (2) determine knowledge gaps for *P. kudriavzevii* and identify research priorities.

## Methods

### Study design

A systematic review was performed as per the Preferred Reporting Items for Systematic Review and Meta‐Analyses (PRISMA) guidelines.

### Inclusion and exclusion criteria

The criteria used to assess features and global impact of IFD caused by *P. kudriavzevii (C. krusei)* were mortality, hospitalisation, disability, antifungal drug resistance, preventability, yearly incidence, global distribution, and emergence in the last 10 years. To ensure a comprehensive analysis, the chosen criteria encompass various aspects of disease burden and epidemiology. Studies were considered for inclusion if they satisfied the following criteria: (1) patient population included adults and/or paediatric patients, (2) included data on *P. kudriavzevii*, (3) included data on at least one criterion for the prioritisation (i.e., study measure), (4) were retrospective or prospective observational studies, randomised controlled trials, epidemiology or surveillance reports, and (5) articles had to be published within the last 10 years (1 January 2011 to 18 February 2021). Studies were excluded if reported on: (1) non-human data, (2) non-fungal data, (3) no data on the selected criteria, (4) <50 patients or isolates, (5) novel antifungal agents (in pre-clinical, early phase trials or not licenced), (6) novel diagnostic tools (not registered for routine clinical use), (7) *in vitro* studies of resistance mechanism(s), (8) case reports, conferences, abstracts, or reviews, (9) articles not written in English, and (10) articles published outside the study period.

### Search strategy

PubMed and Web of Science databases were searched for possibly eligible studies published from 1 January 2011 to 18 February 2021. On PubMed, the search was optimised using the medical subject headings (MeSH) and/or keyword terms in the title/abstract for *P. kudriavzevii (C. krusei)* and criterion. The final search used was (*C. krusei* [Title/Abstract]) combined, using AND term, with criteria terms including (mortality [MeSH Terms]) OR (morbidity [MeSH Terms]) OR (hospitalisation [MeSH Terms]) OR (disability[All Fields])) OR (drug resistance, fungal[MeSH Terms]) OR (prevention and control[MeSH Subheading]) OR (disease transmission, infectious[MeSH Terms]) OR (diagnostic[Title/Abstract]) OR (antifungal agents[MeSH Terms]) OR (epidemiology[MeSH Terms]) OR (surveillance [Title/Abstract]).

On Web of Science, MeSH terms are not available, and therefore a topic search (TS), title search (TI), or abstract (AB) search was used. The final search used [TI=(‘*Candida krusei*’) OR AB=(‘*Candida krusei*’)], combined, using AND term, with criteria terms each as topic search, including (mortality) OR (case fatality) OR (morbidity) OR (hospitali*ation) OR (disability) OR (drug resistance) OR (prevention and control) OR (disease transmission) OR (diagnostic) OR (antifungal agents) OR (epidemiology) OR (surveillance).

PubMed and Web of Science databases are underpinned by a standardised taxonomy database,^8^ and therefore search terms using a species name will also retrieve articles where updated or obsolete nomenclature have been used. Hence, searches using the *Candida krusei* term retrieved articles utilising either *C. krusei* or *P. kudriavzevii*.

### Study selection

Articles searched from each database were imported into a reference manager, EndNote®. These search results were assessed using the online systematic review software, Covidence® (Veritas Health Innovation, Australia). Duplicate publications were removed. The remaining articles underwent title and abstract screening based on the inclusion criteria. The reasons for excluding articles were recorded during full text screening. The title/abstract screening and full text screenings were performed independently by two reviewers (HYK and SLS). Discrepancies were resolved by a third reviewer (JWA).

### Data extraction

Data from the included studies were extracted for each relevant criterion by one reviewer (HYK) and independently checked by a second reviewer (JB).

### Risk of bias assessment

The risk of bias assessment was independently performed by two reviewers (HYK and JB) for the included studies on relevant bias criteria, depending on the study design. Risk of bias tool for randomised trials version 2 (ROB 2) tool and Risk of bias in non-randomised studies (RoBANS) tool were used to assess the randomised controlled trials and non-randomised trials, respectively.^9,10^ The studies were rated as low, high, or unclear risk. Each outcome criterion was assessed if any bias was expected based on the study design, data collection, or analysis in that particular study for the selected outcomes.

### Data synthesis

The extracted data on the outcome criteria were quantitatively or qualitatively synthesised depending on the amount and nature of the data. Data synthesis was performed independently by two reviewers (HYK and JB).

## Results

### Study selection

**Figure 1.**
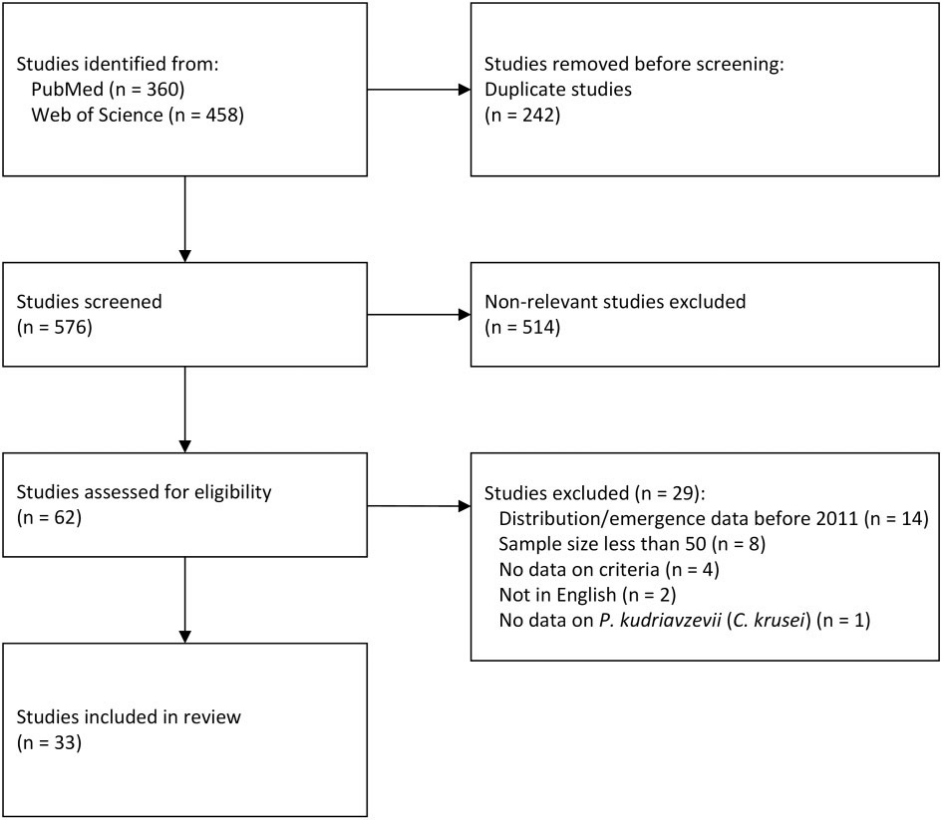
PRISMA flow diagram for selection of studies included in the systematic review of *P. kudriavzevii. Based on:* Preferred Reporting Items for Systematic Reviews and Meta-Analyses: The PRISMA Statement.

Overall, 818 articles were identified in PubMed (*n* = 360) and the Web of Science Core Collection (*n* = 458) databases. After excluding duplicated and non-relevant articles, 62 articles underwent full text screening of which 33 articles were included in the final analysis (Fig. 1).

### Risk of bias

Of the included studies, 18 studies were classified as low risk of bias in all domains assessed (Table 1). Fifteen studies were classified as unclear risk of bias, due to confounding variables and selection biases caused by unclear eligibility criteria or population groups.

**Table 1.** Risk of bias of included studies.

Author	Year	Risk level
Arendrup^27^	2013	Low
Arikan-Akdagli^75^	2019	Unclear
Awad^76^	2018	Unclear
Badiee^19^	2017	Unclear
Bassetti^11^	2011	Low
Castanheira^28^	2020	Low
Castanheira^29^	2014	Low
Castanheira^24^	2014	Unclear
Chen^77^	2017	Unclear
Desnos-Ollivier^78^	2019	Unclear
Fuller^36^	2019	Low
Seyoum^26^	2020	Unclear
Hrabovsky^79^	2017	Low
Israel^21^	2019	Low
Jung^30^	2020	Low
Kakeya^31^	2018	Unclear
Kaur^12^	2020	Low
Kaur^23^	2020	Unclear
Kronen^13^	2018	Low
Lausch^32^	2018	Low
Omrani^14^	2014	Unclear
Orasch^33^	2018	Low
Pfaller^80^	2011	Unclear
Pfaller^25^	2015	Unclear
Puig-Asensio^34^	2014	Low
Salse^81^	2019	Unclear
Sasso^37^	2017	Low
Siopi^35^	2020	Low
Tóth^22^	2019	Unclear
van Schalkwyk^15^	2018	Low
Yacoub^16^	2016	Low
Yang^82^	2018	Unclear
Zeng^83^	2019	Low

### Deaths

Six studies reported on the mortality related to *P. kudriavzevii*. Mortality in adult patients with candidaemia ranged from 44 to 67% (Table 2).^11–14^ Mortality for paediatric patients ranged from 14.6 to 22.94%.^12,15^ One study reported a mortality rate of 19.23% in cancer patients with candidaemia.^16^ The predictors for mortality in *P. kudriavzevii* infected patients were reported to include neutropenia (neutrophil count < 500/mm^3^), lymphoma, prior glucocorticoid use, chronic liver disease, and elevated creatinine (>1 mg/dl or 88.4 mmol/l) (all *P* < 0.05).^13^

**Table 2.** Mortality associated with *P. kudriavzevii*.

Author	Year	Study design		Study period	Country	Level of care	Population description	Number of patients	Number of *P. kudriavzevii* infected patients	Mortality (type, n/n, %)
Bassetti^11^	2011	Prospective cohort study	Single centre	01/2008-12/2010	Italy	Tertiary	Patients with candidaemia	348	9	5/9 (55.5%)
Kaur^12^	2020	Retrospective cohort study	Single centre	01/2014-12/2014	India	Tertiary	Adult and paediatric patients with candidaemia	316 (*n* = 186 paediatric, 130 adults)	316	Paediatric patients:17/74 (22.94%), adult patients: not reported
Kronen^13^	2018	Retrospective cohort study	Single centre	01/2002-01/2015	United States	Tertiary	Patients with candidaemia	1873	59	90-day all-cause mortality for bloodstream infection (BSI): 64.40%
Omrani^14^	2014	Retrospective cohort study	Single centre	01/2003-12/2012	Saudi Arabia	Tertiary	Patients with invasive *Candida* infections	652	9	30-day mortality: 4/9 (44%), 90-day mortality: 6/9 (67%)
van Schalkwyk^15^	2018	Retrospective cohort study	Single centre	01/2012-12/2016	South Africa	Tertiary	Neonates with bloodstream infections during multiple outbreaks	589 during the first outbreak	48	7/48 (14.6%)
Yacoub^16^	2016	Retrospective cohort study	Single centre	01/2001-06/2014	United States	Tertiary	Cancer patients with candidaemia	247	32	19.23%

### Inpatient care

Only one study in a tertiary care centre conducted in neonates with bloodstream infections (BSI) reported a median (IQR) length of hospital stay of 39 days (IQR 25–55) for those infected with *P. kudriavzevii*.^15^ The length of stay for neonates with *P. kudriavzevii* candidaemia was significantly longer than for those with non-*P. kudriavzevii* candidaemia (7 days, IQR 1–17, *P* < 0.001).

### Antifungal resistance

In total, 20 studies reported drug susceptibility data on *P. kudriavzevii* (Table 3). Of these, 13 studies were conducted in patients with invasive fungal infections or candidaemia specifically. Only Kaur et al. (2020) reported drug susceptibility of *P. kudriavzevii* in paediatric patients in India.^12^

**Table 3.** Studies reporting drug susceptibility of *P. kudriavzevii*.

Author	Year	Study design		Study period	Country	Level of care	Population description	Number of patients	Number of isolates	Number of *P. kudriavzevii* isolates	Samples collected from
Arendrup^27^	2013	Prospective national surveillance study	Multi-centre	2010-2011	Denmark	Tertiary	Patients with fungaemia	995	1081 fungal isolates	52	Blood
Arikan-Akdagli^75^	2019	Retrospective cohort study	Multi-centre	1997-2017	Turkey	Tertiary	*Candida* spp. isolates from 12 centres	ND	1991 *Candida* spp.	52	Blood
Badiee^19^	2017	Cross sectional study	Multi-centre	2014-2015	Iran	Tertiary	Immunocompromised patients admitted to 10 hospitals in Iran	ND	846 *Candida* spp.	23	Various sites (blood, CSF, bronchoalveolar lavage, and sputum)
Castanheira^28^	2020	Global surveillance study	Multi-centre	01/2016-12/2017	Asia Pacific, Europe, Latin America, North America	Tertiary	Patients with *Candida* infections (from 60 hospitals in 25 countries)	2936	2936 *Candida* spp.	76	Various sites (majority blood)
Castanheira^29^	2014	Global surveillance study	Multi-centre	2012	Europe, Latin America, North America and the Asia-Pacific Region	Tertiary	Patients with invasive fungal infections	ND	1717	36	Various (blood, sterile body fluids, tissues, abscesses, respiratory tract)
Castanheira^24^	2014	Cross sectional study	Multi-centre	2012	North America, Europe, Latin America, and the Asia Pacific region	Tertiary	Patients with *Candida* spp. infection (from 75 medical centres globally)	ND	1421	32	Various (blood, sterile body fluids, tissues, abscesses)
Chen^77^	2017	Retrospective cohort study	Single centre	01/2007-12/2012	Taiwan	Tertiary	Patients with candidaemia	ND	709 *Candida* spp.	13	Blood
Desnos-Ollivier^78^	2019	Retrospective cohort study	Multi-centre	01/2015-10/2017	France	Tertiary	Patients with invasive infections	ND	1457	76	Blood (majority), CSF, and other
Fuller^36^	2019	Prospective cohort study	Multi-centre	01/2011-10/2016	Canada	Tertiary	Patients with bloodstream infections	ND	1882 *Candida* spp.	81	Blood
Seyoum^26^	2020	Retrospective cohort study	Multi-centre	01/2018-09/2018	Ethiopia		Patients with yeast isolated	ND	209 yeast	14	
Hrabovsky^79^	2017	Retrospective cohort study	Single centre	01/2013-06/2015	Slovakia	Tertiary	Adult non-neutropenic ICU patients	426	800 yeasts	69	Sterile (*n* = 101), non-sterile body sites (*n* = 699)
Israel^21^	2019	Retrospective cohort study	Multi-centre	01/2005-12/2016	Israel	Tertiary and secondary	Patients with candidaemia	899	919 *Candida* spp.	54	Blood
Kaur^12^	2020	Retrospective cohort study	Single centre	01/2014-12/2014	India	Tertiary	Adult and paediatric patients with candidaemia	316 (n = 186 paediatric, 130 adults)	316 *Candida* spp.	96	Blood
Kaur^23^	2020	Retrospective cohort study	Single centre	01/1999-12/2018	India	Tertiary	Patients with candidaemia	7927	7927	527	Blood
Omrani^14^	2014	Retrospective cohort study	Single centre	01/2003-12/2012	Saudi Arabia	Tertiary	Patients with invasive *Candida* infections	652	800 *Candida* spp.	9	Sterile sites (blood, CSF, other body fluid, tissue biopsies)
Pfaller^80^	2011	Retrospective cohort study	Multi-centre	01/2008-12/2009	Asia-Pacific (16 centres, 51 isolates), European (25 centres, 750 isolates), Latin American (10 centres, 348 isolates) and North American (28 centres, 936 isolates) regions.	Tertiary	Patients with candidaemia reported under global surveillance	1752	1752	36	Blood
Pfaller^25^	2015	Retrospective cohort study	Multi-centre	2013	North America (695 isolates, 29 sites), Europe (511 isolates, 19 sites), the Asia-Pacific region (222 isolates, 12 sites), and Latin America (185 isolates, 10sites).	Tertiary	Patients with invasive fungal infections	1320	1320 *Candida* spp.	37	Blood (majority), sterile body fluids (CSF, pleural and peritoneal fluids), tissues, abscesses, respiratory tract and other
Salse^81^	2019	Retrospective cohort study	Multi-centre	2004-2018	France	Tertiary	Patients with infections by yeast and *Aspergillus fumigatus* species from 12 French hospitals	ND	575	575	Blood, sterile sites and other sites, such as bronchoalveolar lavage, sputum
Sasso^37^	2017	Retrospective cohort study	Single centre	2007-2016	France	Tertiary	ICU patients with invasive *Candida* infections	244	3557	192	Blood, other sterile sites
Tóth^22^	2019	Retrospective cohort study	Single centre	01/2005-12/2018	Hungary	Tertiary	Patients with *P. kudriavzevii* isolates collected	53	53	53	Sterile body sites (blood, cerebrospinal, pleural and peritoneal fluids, deep wounds, etc.)

CSF=cerebrospinal fluid, ND=no data, ICU=intensive care unit.

Clinical breakpoints are only available for some antifungals in *P. kudriavzevii* (amphotericin B and anidulafungin in the case of EUCAT AFST). When not available, epidemiological cutoff values, abbreviated ECVs (CLSI—the Clinical and Laboratory Standards Institute) or ECOFFs (EUCAST—the European Committee on Antimicrobial Susceptibility Testing), were used to classify an isolate as wild-type (WT) or non-wild-type (non-WT).^17,18^ In studies assessing susceptibility of *P. kudriavzevii* of azoles other than fluconazole, most (n/n = 17/19, 89.5%) reported low resistance or non-WT rates, ranging from 0 to 5.6% (Tables 4 and 5). However, two studies (2/19, 10.5%) found higher resistance rates for (33.3–71.2%), voriconazole (20–88.5%), and posaconazole (96.2%).^19,20^ For echinocandins, most studies reported low non-WT rates of 0–5% (Table 5). However, in three studies (3/16, 18.8%), higher non-WT rates of 30–67% were reported for caspofungin.^19,21,22^ Resistance to amphotericin B was also low ranging from 0 to 5%, with the exception of two studies (2/12, 16.7%) reporting rates of 12.9 and 40%.^19,23^ For flucytosine, two studies reported *P. kudriavzevii* is wild-type but one reported a non-WT rate of 78.6%.^24–26^

**Table 4.** Drug susceptibility of *P. kudriavzevii* to azoles.

Author	Year	MIC method	Fluconazole	Isavuconazole	Itraconazole	Posaconazole	Voriconazole
Arendrup^27^	2013	EUCAST (EUCAST BP, CLSI BP for itraconazole)	(*n* = 52) 0% S	ND	(*n* = 52) 28.8% S	(*n* = 52) 3.8% S	(*n* = 52) 11.5% S
Arikan-Akdagli^75^	2019	CLSI	(*n* = 52) GM MIC (range): 27.64 (8- >64), 100% R	ND	0.17 (≤0.015-0.5), 0% non-WT	0.14 (≤0.03-1), 1.9% non-WT	0.07 (0.03-0.125), 100% S
Badiee^19^	2017	CLSI (susceptibility based on CLSI BP, or ECV)	GM MIC (range): 17.9 (2-64), >64 (5%) non-WT	ND	0.2 (0.064-1), 33.3% R	0.126 (0.032-0.5), >0.5 (5%) non-WT	0.284 (0.032-16), 20% R
Castanheira^28^	2020	CLSI	ND	ND	ND	0% non-WT	1.3% R (5% R in North America, *n* = 20)
Castanheira^29^	2014	CLSI	ND	ND	ND	5.6% R	2.8% R
Castanheira^24^	2014	CLSI	ND	MIC/MEC range: 0.12-2, MIC/MEC_50_: 0.5, MIC/MEC_90_: 0.5, % not available	0.25-4, 0.25, 0.5, 3.1% non-WT	0.12-2, 0.25, 0.5, 6.3% non-WT	0.12-4, 0.25, 0.25, 3.1% non-WT
Chen^77^	2017	Sensititre YeastOne	MIC range: 32-128, MIC_50_: 64, MIC_90_: 64 Considered intrinsically resistant	ND	ND	ND	0.12-0.5, 0.5, 0.5, 0% R 100% S
Desnos-Ollivier^78^	2019	EUCAST	MIC range:16- ≥64, MIC_50_: 32, MIC_90_: 64 % R not available (considered intrinsically resistant)	MIC range:0.015-1, MIC_50_: 0.125, MIC_90_: 0.25 %isolates with MIC>MIC_90_: 6.58%	ND	ND	ND
Fuller^36^	2019	CLSI	mode MIC: 8, MIC_90_: 16	ND	ND	ND	MIC_90_: 0.25
Seyoum^26^	2020	VITEK 2 compact system	*n* = 14, 100% R	ND	ND	ND	0% R
Hrabovsky^79^	2017	EUCAST	(*n* = 40 isolates for invasive disease) MIC range: 2-256, MIC_50_: 256, MIC_90_: 256 100% R	ND	ND	ND	0.094-4, 0.5, 1,5% R
Israel^21^	2019	CLSI	NA (considered intrinsically resistant)	ND	ND	ND	(*n* = 54), 3.8% R
Kaur^12^	2020	CLSI	ND	ND	(*n* = 82 paediatric isolates) GM MIC (range): 0.31 (0.12-0.5), MIC_50_: 0.25, MIC_90_: 0.5	0.24 (0.06-0.5), 0.25, 0.5	0.41 (0.05-8), 0.25, 0.25
Kaur^23^	2020	CLSI	For 2014-2018 period: 40.5% R	ND	4.2% R	0% R	1.9% R
Omrani^14^	2014	CLSI	*n* = 13, 0% S	ND	ND	ND	*n* = 6, 100% S
Pfaller^80^	2011	CLSI	ND	ND	ND	*n* = 16 ICU, 0% R, n = 20 non-ICU, 0% R	*n* = 16 ICU, 0% R, n = 20 non-ICU, 0% R
Pfaller^25^	2015	CLSI	MIC/MEC range: 8- >128, MIC_50_: 32, MIC_90_: 64, Intrinsically resistant.	MIC/MEC range: 0.12-4, MIC_50_: 0.5, MIC_90_: 1	MIC/MEC range: 0.25-2, MIC_50_: 0.5, MIC_90_: 1, 2.7% non-WT, 97.3% WT	MIC/MEC range: 0.25-1, MIC_50_: 0.5, MIC_90_: 0.5,2.7% non-WT, 97.3% WT	2.7%R, 94.6% S
Salse^81^	2019	E-test	*n* = 414, mode MIC: >256	ND	ND	ND	*n* = 575, mode MIC: 0.5
Sasso^37^	2017	E-test (CLSI BP)	100% R (*n* = 48) (averaged for 2007-2016)	ND	ND	ND	79.4% S (*n* = 55) 29.6% I (*n* = 47) (averaged for 2007–2016)
Tóth^22^	2019	CLSI	mode MIC (range): 32 (8- >32), MIC_50_: 32, MIC_90_: >32%, R ND	ND	ND	ND	ND

Data are reported as they appear in source documents. Susceptibility is expressed as mg/l unless indicated otherwise. BP=breakpoint, CLSI=Clinical and Laboratory Standards Institute, ECV=epidemiological cutoff value, EUCAST= European Committee on Antimicrobial Susceptibility Testing, R=resistant, S=susceptible, S-DD=susceptible dose-dependent, I=intermediate, MIC= minimum inhibitory concentration, MEC=minimum effective concentration, GM= geometric mean, NA/ND= not applicable / not done, MIC_50_=MIC required to inhibit the growth of 50% of isolates, MIC_90_=MIC required to inhibit the growth of 90% of isolates, ND= no data, Non-WT = non wild-type.

**Table 5.** Drug susceptibility of *P. kudriavzevii* to non-azole antifungal drugs.

Author	Year	MIC method	Anidulafungin	Caspofungin	Micafungin	Amphotericin B	Flucytosine
Arendrup^27^	2013	EUCAST (EUCAST BP, CLSI BP for caspofungin and itraconazole)	(*n* = 52) 100% S	(*n* = 25) 28% S	ND	(*n* = 52) 73.1% S	ND
Arikan-Akdagli^75^	2019	CLSI	ND	ND	0.08 (≤0.03-0.25), % S	1.32 (0.5-2), 0% non-WT	ND
Badiee^19^	2017	CLSI (susceptibility based on CLSI BP, or ECV)	ND	0.2 (0.032-2), 30% R	ND	1.004 (0.032-8), 40% R	ND
Castanheira^28^	2020	CLSI	0% R	0% R	0% R	0% non-WT	ND
Castanheira^29^	2014	CLSI	2.8% R	2.8% R	0% R	ND	ND
Castanheira^24^	2014	CLSI	0.03-1, 0.06, 0.12, 3.1% non-WT	0.06-1, 0.12, 0.25, 3.1% non-WT	0.015-0.12, 0.12, 0.12, 0% non-WT	1-2,1,2,0% non-WT	8-32, 16, 16, 0% non-WT
Chen^77^	2017	Sensititre YeastOne	0.12-0.25, 0.12, 0.12, 0% R 100% S	0.25-0.5, 0.5, 0.5, 0% R, 23.1%S, 76.9% I	0.6-0.12, 0.12, 0.12, 0% R, 100% S	ND	ND
Fuller^36^	2019	CLSI	ND	MIC not available, 0% R	MIC not available, 0% R	MIC not available, 100% WT (based on ECV ≤2)	ND
Seyoum^26^	2020	VITEK 2 compact system	ND	0% R	0% R	ND	78.6% R
Hrabovsky^79^	2017	EUCAST	0.002-0.19, 0.008, 0.023,5% R	0.002-0.25, 0.063,0.125, %R ND	ND	0.19-2, 0.5,1,5% R	ND
Israel^21^	2019	E-test (CLSI BP)	ND	67% R	ND	1.9% R	ND
Kaur^12^	2020	CLSI	0.28 (0.03-4), 0.12, 0.5	0.35 (0.12-2), 0.12, 0.5	0.45 (0.06-12), 0.12,0.5	0.90 (0.25-2),1,1	ND
Kaur^23^	2020	CLSI	1.9% R	16% R	2.5% R	12.9% R	ND
Omrani^14^	2014	CLSI	ND	*n* = 6, 66.7% S	ND	*n* = 14, 100% S	ND
Pfaller^80^	2011	CLSI	*n* = 16 ICU, 0% R, n = 20 non-ICU, 0% R	*n* = 16 ICU, 6.3% R, n = 20 non-ICU, 5.0% R	*n* = 16 ICU, 0% R, n = 20 non-ICU, 0% R	ND	ND
Pfaller^25^	2015	CLSI	0% R, 100% S	0% R, 100% S	0% R, 100% S	MIC/MEC range: 1-2, MIC_50_: 1, MIC_90_: 2,0% non-WT, 100% WT	MIC/MEC range: 8-32, MIC_50_: 16, MIC_90_: 32, 0% non-WT, 100% WT
Salse^81^	2019	E-test	*n* = 117, mode MIC: 0.03	*n* = 565, mode MIC: 0.5	*n* = 259, mode MIC: 0.25	*n* = 534, mode MIC: 1	ND
Sasso^37^	2017	E-test (CLSI BP)	ND	62.6% S (*n* = 31) 86.8% I (*n* = 50) (averaged for 2007-2016)	ND	100% WT (*n* = 51)	ND
Tóth^22^	2019	CLSI	0.06 (0.015-0.25), 0.06, 0.12,100% S	1 (0.12-1), 1,1,11.3% S, 22.6% I, 66.1% R	0.25, (0.03-0.25), 0.25,0.25, 100% S	1 (0.5-2),1,1	ND

Data are reported as they appear in source documents. Susceptibility is expressed as mg/l unless indicated otherwise.

BP=breakpoint, CLSI= Clinical and Laboratory Standards Institute, ECV=epidemiological cutoff value, EUCAST= European Committee on Antimicrobial Susceptibility Testing, R=resistant, S=susceptible, S-DD=susceptible dose-dependent, I=intermediate, ICU=intensive care unit, MIC= minimum inhibitory concentration, MEC=minimum effective concentration, GM= geometric mean, NA/ND= not applicable / not done, MIC_50_=MIC required to inhibit the growth of 50% of isolates, MIC_90_=MIC required to inhibit the growth of 90% of isolates, ND= no data, Non-WT = non wild-type.

### Preventability of infection

Five studies reported on risk factors for infection by *P. kudriavzevii* (Table 6). The prevalence of this species among candidaemias was reportedly higher in paediatric patients compared to adults (44–80% in paediatric vs. 11–20% in adult patients, *P* < 0.001).^12,23^ Neonates weighed <2 kg were significantly more likely to have a BSI as compared with those who weighed >2.5 kg (adjusted odds ratio [aOR] 3.4–6.1, *P* < 0.05).^15^ Extremely low body weight of <1 kg was associated with an even greater OR of 6.5 in this analysis (*P* = 0.002).^15^ Neonates with necrotiseng enterocolitis were also at risk of *P. kudriavzevii* (aOR = 3.1, *P* = 0.005).^15^ In adults, malignancy (especially haematologic and gastric: OR of 10.7 and 14.7, *P* < 0.05), neutropenia (OR = 2.1, *P* = 0.027), prior use of azole antifungal drugs (OR = 2.4, *P* = 0.013), monoclonal antibodies (i.e., antilymphocyte, antimyeloid, anti-TNF; OR = 5.4, *P* = 0.001), or β-lactam/β-lactamase inhibitors (OR = 2.4, *P* = 0.009), were associated with an increased risk for *P. kudriavzevii*.^13^ Hand hygiene intensification and subsequent increase in hygiene compliance (from 58.51% in 2014 to 73.2% in 2016) on paediatric wards in an Indian tertiary hospital correlated with a decline of *P. kudriavzevii* candidaemia from 44.09 to 6.97%.^12^

**Table 6.** Risk factors for infections caused by *P. kudriavzevii*.

Author	Year	Study design		Study period	Country	Level of care	Population description	Number of patients	Number of *P. kudriavzevii* isolates	Risk factors
Kaur^12^	2020	Retrospective cohort study	Single centre	01/2014-12/2014	India	Tertiary	Adult and paediatric patients with candidaemia	316 (*n* = 186 paediatric, 130 adults)	316	Significantly greater prevalence in paediatric group (44%, 82/186) vs. adults (10.8%, 14/130; *P* < 0.001).Gastrointestinal disease (*P* = 0.018), prior use of antibiotics (*P* = 0.021), exposure to carbapenems (*P* = 0.039).
Kaur^23^	2020	Retrospective cohort study	Single centre	01/1999-12/2018	India	Tertiary	Patients with candidaemia	7927	527	Paediatric patients: 422/527 (80.1%) paediatric vs. 105/527 (19.9%) adults
Kronen^13^	2018	Retrospective cohort study	Single centre	01/2002-01/2015	US	Tertiary	Patients with candidaemia	1873	59	Six variables (multivariate analysis): Haematologic malignancy (OR, 10.7; 95% CI, 5.1-22.4),gastric malignancy (OR, 14.7; 95% CI, 3.0-72.8), neutropenia (OR, 2.1; 95% CI, 1.1-4.1), prior azole use (OR, 2.4; 95% CI, 1.2-4.7), prior monoclonal antibody use (OR, 5.4; 95% CI, 2.0-14.9), and β-lactam/β-lactamase inhibitor use (OR, 2.4; 95% CI, 1.3-4.7) within 90 days prior to *Candida* BSI.
Lausch^32^	2018	Retrospective cohort study	Multi-centre	2010-2011	Denmark	Mixed (data from national surveillance)	Adult patients with candidaemia	841	35	Prior antifungal treatment (AFT): Substantially higher in patients with prior AFT ([12.9% for azoles and 9.1% for echinocandins] vs. 2.2% without prior AFT)
van Schalkwyk^15^	2018	Retrospective cohort study	Single centre	01/2012-12/2016	South Africa	Tertiary	Neonates with blood-stream infections during multiple outbreaks	589 during the first outbreak	48	With *P. kudriavzevii* candidaemia vs. without: Necrotising enterocolitis (aOR 3.1, 95%CI 1.4-6.7),Birthweight (in reference to >2.5kg):extreme low <1kg (aOR 6.5, 95%CI 1.9-21.6),1- <1.5 kg (6.1 (2.1-17.2)),1.5-1.9 kg (3.4 (1.1-10.0))

AFT=antifungal treatment, aOR=adjusted odds ratio, OR=odds ratio, BSI=bloodstream infection.

### Annual incidence of infections

A prospective national surveillance study in Denmark reported an incidence of *P. kudriavzevii of* 0.45 per 100 000 inhabitants during 2010–2011.^27^ The study was conducted in 13 tertiary care centres and found a stable incidence rate of *P. kudriavzevii* infection from 2004 to 2011 (∼5% of all *Candida*-like blood isolates). The low incidence rate reported by this study emphasises the rarity of *P. kudriavzevii* infections.

### Global distribution

**Figure 2.**
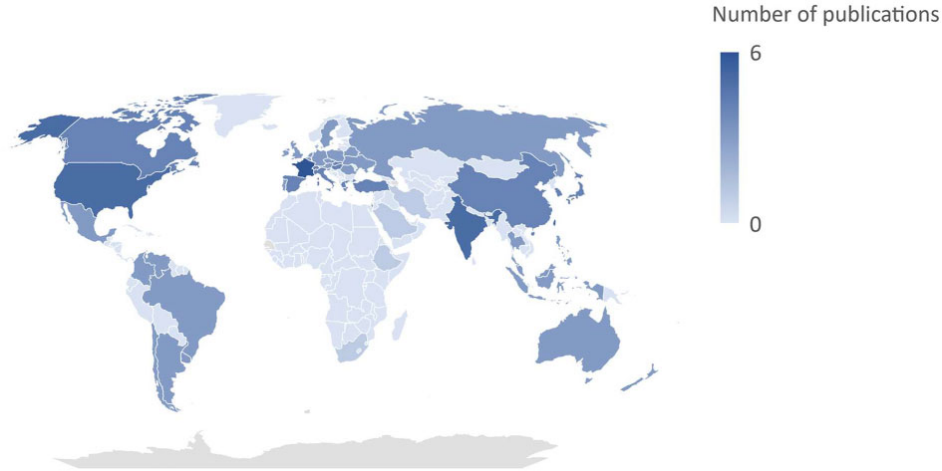
Global distribution of *Pichia kudriavzevii* (formely *Candida krusei*) from 2011 to 2021.

Overall, 26 studies reported data on the distribution and emergence of *P. kudriavzevii* in various regions around the world (Fig. 2; Table S1). Whilst the organism is globally distributed, variations by geographic regions exist. Understanding these geographic variations is crucial for tailoring regional strategies to address *P. kudriavzevii* infections. Due to variable study populations, direct comparison of the distribution of *P. kudriavzevii* between geographic regions was challenging. Global surveillance studies reported *P. kudriavzevii* in 2.6% (*n* = 76/2936) and 2.1% (*n* = 36/1717) of cases, respectively.^28,29^ This is comparable to other studies, generally reporting a low prevalence of candidaemia due to *P. kudriavzevii* among *Candida* species in adults that ranged from 1 to 10.8%.^12,13,21,23,30–36^*Pichia kudriavzevii* was most frequently reported in Europe (46–56%; *n* = 35/76; *n* = 20/36), North America (26–28%; *n* = 20/76; *n* = 10/36), Latin America (8–16%; *n* = 12/76; *n* = 3/36), and the Asia-Pacific region (8–12%; *n* = 9/76, *n* = 3/36) in patients with *Candida* IFD.^28,29^ A high prevalence rate of 44% *P. kudriavzevii* candidaemia in paediatric patients in India related to an outbreak in a single year was reported.^12^ Environmental sources appeared to be a washbasin and genetic similarity with the environmental isolates was demonstrated.

#### Trends in the incidence of infections caused by P. kudriavzevii in the last 10 years

Trends in the incidence of *P. kudriavzevii* were variable over the last 10 years (Table S1). A stable incidence rate of 0–6% was reported in Japan, Denmark, Canada, and Saudi Arabia.^14,27,31,36^ A low overall incidence rate of 1.4–4.3% but with fluctuations during the period of 2011–2014 was reported in the US (2011: 4.3%, 2012: 1.4%, 2013: 4.3%, 2014: 2.1%).^13^ Higher incidence rates (up to 10%) were reported in France and Israel, although overall the incidence rates are decreasing from 9–10% to 2.6–3%.^21,37^ One study in India observed an increased incidence from 5.6% in 2009–2013 to 9.3% in 2014–2018.^23^ One study in the US in cancer patients with candidaemia reported a higher overall rate of 14–15%, which was stable during the study period of 2006–2014.^16^

## Discussion

*Pichia kudriavzevii (C. krusei)* causes severe infections in various organs and tissues including urinary, respiratory, and gastrointestinal tract and bloodstream. This can be explained by its ability to adhere to host tissue and form biofilms. By excreting proteases and phospholipases it damages the host tissue and becomes invasive. Its ability to evade the immune system and persistence in various conditions further increases the infection risk. Infections caused by *P. kudriavzevii* were associated with high mortality rates ranging from 44 to 67%, particularly in adults with haematologic and gastric malignancies (Table 2). The high 90-day all-cause mortality observed in *P. kudriavzevii* candidaemia likely reflects the underlying life-threatening conditions rather than the virulence of the pathogen itself.^13^ The association between *P. kudriavzevii* candidaemia and mortality is less pronounced when accounting for potential confounders such as lymphoma, neutropenia, glucocorticoid use, chronic liver disease, and elevated creatinine concentrations (HR, 1.3; 95% CI, 0.9–1.8 in multivariable analysis *versus* HR, 1.8; 95% CI, 1.3–2.4 in univariable analysis).^13^ This epidemiological evidence is supported by *in vitro* and *in vivo* virulence tests demonstrating that *P. kudriavzevii* is a relatively low-virulence pathogen, i.e., no mortality, no weight loss, no metastatic eye infections, no or discrete kidney inflammation in mice models, compared with other *Candida*-like species.^38^ The mortality rate of *P. kudriavzevii* is lower in paediatric compared to adult populations, which may be due to the severity of co-morbidities in adults. Numerous factors which predict mortality associated with *P. kudriavzevii* have been identified.^6^

Overall, patients with immature/suppressed immune systems or imbalanced bacterial-fungal ecosystem in gut are at an increased risk of infection by *P. kudriavzevii*. The risk factors vary with age which include prior antibiotic/antifungal use. Antibiotics cause long-term imbalance of human gut microbiome where the eradication of certain bacteria makes room for opportunistic fungal pathogens to invade.^40,41^ Similarly, antifungals can cause bacterial-fungal imbalance in mice gut, disrupting healthy symbiotic gut flora and immune homeostasis.^42^ In addition, the overuse or misuse of prophylactic antifungal treatment appears to lead to the selection of inherently less susceptible fungal species.^43–45^ As the transmission of *P. kudriavzevii* is common from the hands of healthcare workers and the healthcare environment,^12,46–48^ reinforcement of hand hygiene practices and maintenance of central venous catheters has been shown to assist with infection control including for *P. kudriavzevii*.^23,49^ Preventative studies based on the identified risk factors should be explored for their potential benefit and feasibility for implementation to prevent *P. kudriavzevii* infections in these at-risk populations, including education,^50^ proper antifungal prophylaxis,^51^ weekly surveillance rectal swabs^52^ and avoidance of unnecessary broad-spectrum antibiotics.^50^

The impact of *P. kudriavzevii* infections on length of hospital stay is poorly understood and requires further research. Clinical experience indicates that *P. kudriavzevii* is unlikely to cause long-term disability and secondary eye infections are rare.^39^

Although *P. kudriavzevii* is considered intrinsically resistant to fluconazole, resistance rates to other azoles, anidulafungin and micafungin were mostly low (0–5%). Resistance to *P. kudriavzevii* can result from various mechanisms. Firstly, mutations in the target enzyme for ergosterol synthesis (lanosterol 14α-demethylase, Erg11 or Cyp51) reduce the binding affinity of azole drugs.^53,54^ Secondly, a lower ergosterol content in the cell membrane of *P. kudriavzevii* can reduce the binding sites for amphotericin B, making it less effective.^54^ Lastly, the upregulation of efflux pumps removes drugs from the cell increasing its resistance further. The responsible efflux pump ABC1 is relevant for azole acquired resistance while the efflux pump ABC2 is associated with innate resistance to fluconazole.^55,56^ Alternative antifungals of fluconazole-resistant *P. kudriavzevii* include voriconazole, itraconazole, echinocandins, amphotericin B (higher dose) and flucytosine.^57–59^ Resistance rate to caspofungin, i.e., the proportion of isolates with MICs above the CLSI breakpoint^60^ (EUCAST breakpoint has not been determined because of high variation in caspofungin MICs),^61^ has varied in the included studies. MIC testing for caspofungin is considered unreliable/non-reproducible by both CLSI and EUCAST. ^61^ Whilst most studies reported relatively low non-WT rates of 0–6%, three studies reported higher non-WT rates of 30–67%.^19,21,22^ The high non-WT rates to caspofungin are likely misclassified as (1) applying the CLSI breakpoint to varying MICs might lead to falsely reporting too many wild-type strains as non-susceptible;^19,22,61^ (2) combining the CLSI breakpoint with E-tests has not been validated,^21,60,62^ and (3) higher MIC ranges obtained by E-tests than by CLSI method that might lead to 67% of the cases being misclassified.^38^ In waiting for validated methods for testing caspofungin, EUCAST recommends using anidulafungin or micafungin as predictors of resistance for echinocandins. ^61^ Similarly, reduced susceptibility to amphotericin B has also been observed, with the proportion of isolates with MICs > CLSI ECV ^17^ being reported in India (13%) and Iran (40%).^19,23^ The reduced susceptibility might be explained the fact that amphotericin B is one of the most common antifungal drugs used in these regions.^19^ It is likely that there will be geographic variability of MICs and it is vital that testing laboratories in LMICs always utilise quality control strains to ensure their results are in accord with international standards.^63^ National and/or international surveillance systems are required to systematically monitor the development of resistance for *P. kudriavzevii*. Data from these systems would support clinicians in making decisions based on information from their local region including epidemiology, antimicrobial resistance, and treatment strategies. In addition, appropriate use of antifungal drugs promoted by timely, accurate diagnosis and susceptibility testing will assist in reducing the risk of resistance development.^64^ To reduce the high mortality associated with *P. kudriavzevii*, traditional phenotypic methods (e.g., colony morphology, biochemical tests or Analytical Profile Index - API) are useful for screening *P. kudriavzevii* to initiate echinocandin or amphotericin B instead of fluconazole. However, specialised methods such as DNA sequencing or MALDI-TOF mass spectrometry provide more accurate and reliable species identification. Indeed, nine studies evaluating the accuracy of different methods for the identification of uncommon *Candida* species including *P. kudriavzevii* found that the accuracy using traditional phenotypic methods ranged from 15–76% versus 75–100% for MALDI-TOF MS or sequencing.^65,66^ Higher accuracy when using traditional methods was achieved by colonising *P. kudriavzevii* on CHROMagar medium and incorporating a specific screening test for *P. kudriavzevii* (e.g., immunoassay Krusei-Color Fumouze®) in replacement with API system.^67,68^ Regional- or country-specific treatment strategies should also be developed due to the diverse *P. kudriavzevii* resistance pattern and resources available.^63^ New drugs like rezafungin,^69^ ibrexafungerp^70^ and oteseconazole^71^ will likely be a valuable addition to the therapeutic armamentarium to treat *P. kudriavzevii* infections as they have demonstrated activity against fluconazole resistant isolates.

The incidence of *P. kudriavzevii*, although globally distributed, varies depending on the population studied and geographical location. Generally, the incidence of *P. kudriavzevii* was low and stable across the globe except for India where the incidence has been increasing over the last 5 years (5.6–9.3%). The increased trend in India may be due to high hospital occupancy and challenges in infection control implementation leading to cross-transmission.^11^ It is also possible that incidence rates of *P. kudriavzevii* in LMICs are underestimated due to difficulties in species-level identification in the absence of mass spectrometry or molecular techniques in routine clinical practice.^72,73^

We acknowledge several limitations in our review. First, publication bias cannot be excluded as few observational studies on the incidence and clinical outcomes of *P. kudriavzevii* infections and laboratory-based studies for susceptibility data from LMICs were retrieved by our search. Studies from under-resourced settings may be smaller scale due to limited financial and human resources, leading to publication bias in favour of better resourced settings.^74^ Second, many studies were retrospective cohort studies where selection bias might have occurred and there might have been an absence of data on potential confounders. Third, language bias cannot be ruled out as we only searched English language literature. Considering these limitations, we interpreted the results cautiously. Although studies published before 2011 were excluded, the outcome criteria assessed are time-sensitive rendering older data less informative.

## Conclusion

Mortality in patients with *P. kudriavzevii (C. krusei)* infection was higher for adults than paediatric populations, particularly those with severe co-morbidities. Rapid identification of *P. kudriavzevii* is vital to administering echinocandins or high doses of amphotericin B as initial treatment. Non-WT rates of azoles and echinocandins was low, except for fluconazole. Risk factors for developing *P. kudriavzevii* infections vary with age, notably low birth weight, prior use of antibiotics/antifungals, and an underlying diagnosis of gastrointestinal disease or cancer. The implementation of stewardship programmes focused on addressing these risk factors should be explored for their benefit and feasibility. Although rare, *P. kudriavzevii* is globally distributed with an apparently higher incidence in India. This highlights the need for continued surveillance efforts and targeted interventions to address *P. kudriavzevii* infections, particularly in regions with higher incidence rates. Due to scarce data on incidence and resistance, stronger and global surveillance systems are required to support clinical decision-making for *P. kudriavzevii*.

## Supplementary Material

myad132_Supplemental_Files
