# Supplementary material for: Pichia kudriavzevii (Candida krusei): A systematic review to inform the World Health Organisation priority list of fungal pathogens
Source: Med Mycol. 2024 Jun 27;62(6):myad132. doi: 10.1093/mmy/myad132 (PMC11210618; doi:10.1093/mmy/myad132)
Supplement: myad132_Supplemental_Files [file myad132_supplemental_files.zip › mm-2023-0240-File005.docx]

Table S1. Distribution and incidence of *P. kudriavzevii* over time*.*

| **Author** | **Year** | **Study design** | | **Study period** | **Country** | **Level of care** | **Population description** | **Number of patients** | **Number of *P. kudriavzevii* isolates** | **Distribution** | **Trends** |
| --- | --- | --- | --- | --- | --- | --- | --- | --- | --- | --- | --- |
| Arendrup^27^ | 2013 | Other: Prospective national surveillance study | Multi-centre | 2010-2011 | Denmark | Tertiary | Patients with fungaemia | 995 | 52 | 4.8% (incidence rate per 100,000 inhabitants in the period 2010-2011): 4.1% (2004-2009), 5.0% (2010), 4.7% (2011) | Stable |
| Awad^76^ | 2018 | Retrospective cohort study | Single centre | 01/2010-12/2015 | Lebanon | Tertiary | *Candida* isolates from microbiology laboratory database | ND | 10 | 10/1377 (0.73%) among *Candida* spp. (10/333 (3%) *P. kudriavzevii* out of non-albicans) | ND |
| Badiee^19^ | 2017 | Cross sectional study | Multi-centre | 2014-2015 | Iran | Tertiary | Immunocompromised patients admitted to 10 hospitals in Iran | ND | 23 | 23/846 (2.7%) among *Candida* spp: colonised (n=13), invasive (n=10) | ND |
| Bassetti^11^ | 2011 | Prospective cohort study | Single centre | 01/2008-12/2010 | Italy | Tertiary | Patients with candidaemia | 348 | 9 | ND | Increasing: 0.01 episodes/1000 hospital admissions (2008), 0.06/1000 (2009), 0.07/1000 (2010) |
| Castanheira^28^ | 2020 | Other: Global surveillance study | Multi-centre | 01/2016-12/2017 | Asia Pacific, Europe, Latin America, North America | Tertiary | Patients with *Candida* infections (from 60 hospitals in 25 countries) | 2936 | 76 | 35/76 (46%) Europe, 20/76 (26.3%) North America, 12/76 (15.8%) Latin America, 9/76 (11.8%) Asia Pacific: among *Candida* spp. | ND |
| Castanheira^29^ | 2014 | Other: Global surveillance study | Multi-centre | 2012 | Europe, Latin America, North America and the Asia-Pacific Region | Tertiary | Patients with IFI | ND | 36 | 20/36 (55.6%) Europe,  10/36 (27.8%) North America, 3/36 (8.3%) Latin America,  3/36 (8.3%) Asia-Pacific Region: among IFI | ND |
| Fuller^36^ | 2019 | Prospective cohort study | Multi-centre | 01/2011-10/2016 | Canada | Tertiary | Patients with bloodstream infections | ND | 81 | 81/1882 (4.3%) among *Candida* spp. (2011-2016) | Relatively stable^1^ 2011: 5% 2012: 2% 2013-15: 4-5% 2016: 6% |
| Seyoum^26^ | 2020 | Retrospective cohort study | Multi-centre | 01/2018-09/2018 | Ethiopia |  | Patients with yeast isolated | ND | 14 | 14/194 (7.2%) (*P. kudriavzevii* /*Candida* spp.) (15.6% among NAC species) | ND |
| Hrabovsky^79^ | 2017 | Retrospective cohort study | Single centre | 01/2013-06/2015 | Slovakia | Tertiary | Adult non-neutropenic ICU patients | 426 | 69 | 69/800 (8.6%) among yeasts | ND |
| Israel^21^ | 2019 | Retrospective cohort study | Multi-centre | 01/2005-12/2016 | Israel | Tertiary and secondary | Patients with candidaemia | 899 | 54 | 54/919 (5.9%) among *Candida* spp. | Decreasing pattern 2011: 2.5%, 2012: 10%, 2013: 9%, 2014: 6%, 2015: 5%, 2016: 3% (estimated from the graph) |
| Jung^30^ | 2020 | Retrospective cohort study | Multi-centre | 2012-2015 | South Korea | Tertiary | Adult patients with candidaemia | 317 | 8 | 8/317 (2.5%) among *Candida* spp. | ND |
| Kakeya^31^ | 2018 | Retrospective cohort study | Multi-centre | 2003-2014 | Japan | Tertiary | *Candida* spp. blood culture results from 10 hospitals | ND | 61 | 61/1921 (3.2%) among *Candida* spp. | Stable^2^. 2011-2013: 2.5%, 2014: 3.5% |
| Kaur^12^ | 2020 | Retrospective cohort study | Single centre | 01/2014-12/2014 | India | Tertiary | Adult and paediatric patients with candidaemia | 316 (n=186 paediatric, 130 adults) | 96 | 96/316 (30.3%) overall:  82/186 (44%) among paediatric patients, 14/130 (10.8%) among adults. | ND |
| Kaur^23^ | 2020 | Retrospective cohort study | Single centre | 01/1999-12/2018 | India | Tertiary | Patients with candidaemia | 7927 | 527 | 527/7927 (6.7%) *P. kudriavzevii* out of all candidaemia | Increasing (P<0.05), 2009-2013: 5.6%, 2014-2018: 9.3% |
| Kronen^13^ | 2018 | Retrospective cohort study | Single centre | 01/2002-01/2015 | US | Tertiary | Patients with candidaemia | 1873 | 59 | 59/1873 (3.2%) | Fluctuating but relatively low^3^ 2011: 4.3%, 2012: 1.4%, 2013: 4.3%, 2014: 2.1% |
| Lausch^32^ | 2018 | Retrospective cohort study | Multi-centre | 2010-2011 | Denmark | Mixed (data from national surveillance) | Adult patients with candidaemia | 841 | 35 | 35/841 (4.2%) | ND |
| Omrani^14^ | 2014 | Retrospective cohort study | Single centre | 01/2003-12/2012 | Saudi Arabia | Tertiary | Patients with invasive *Candida* infections | 652 | 9 | 9/652 (1.4%) | Stable 2009: 0/80 (0%) 2010: 2/108 (1.9%) 2011: 2/93 (2.2%), 2012: 0/76 (0%) |
| Orasch^33^ | 2018 | Prospective cohort study | Multi-centre | 2004-2006 | Switzerland | Tertiary | Patients with candidaemia | 567 | 13 | 13/567 (2.3%) among candidaemia: [n=5/43 (12%) associated with breakthrough candidaemia (BTC), n=8/507 (2%) with non-BTC] | ND |
| Pfaller^25^ | 2015 | Retrospective cohort study | Multi-centre | 2013 | North America (695 isolates), Europe (511 isolates), the Asia-Pacific region (222 isolates), and Latin America (185 isolates). | Tertiary | Patients with IFI | 1320 | 37 | North America: 21/37, Europe: 9/37, Latin America: 3/37, Asia-Pacific: 4/37 | ND |
| Puig-Asensio^34^ | 2014 | Prospective cohort study | Multi-centre | 05/2010-04/2011 | Spain | Tertiary | Adult ICU patients with candidaemia | 168 | 7 | 7/168 (4%) among ICU candidaemia | ND |
| Sasso^37^ | 2017 | Retrospective cohort study | Single centre | 2007-2016 | France | Tertiary | ICU patients with invasive *Candida* infections | 244 | 192 | ND | n=3557 isolates 2011: 5.1%, 2012: 9.1%, 2013: 8%, 2014: 4.5%, 2015: 6.3%, 2016: 2.6% |
| Siopi^35^ | 2020 | Other: Retrospective surveillance study + literature review (excluded from analysis) | Single centre | 2009-2018 | Greece | Tertiary | Patients with candidaemia | 429 | 5 | 5/449 (1%) among *Candida* spp. | Variable but low^4^ 0-5%: 2011: 0%, 2012: 3%, 2013-2014: 0%, 2015: 5%, 2016-2018: 0% |
| van Schalkwyk^15^ | 2018 | Retrospective cohort study | Single centre | 01/2012-12/2016 | South Africa | Tertiary | Neonates with bloodstream infections during multiple outbreaks | 589 during the first outbreak | 48 | 01/2012-12/2016: 91/262 (35%) among candidaemia including period of multiple outbreaks,  First outbreak 07/2014-10/2014: 48/589 (8.2%) *P. kudriavzevii* /neonatal admissions | ND |
| Yacoub^16^ | 2016 | Retrospective cohort study | Single centre | 01/2001-06/2014 | US | Tertiary | Cancer patients with candidaemia | 247 | 32 | ND | 2006-2010: 14% in cancer patients 2011-2014: 15% |
| Yang^82^ | 2018 | Retrospective cohort study | Multi-centre | 2014 | Taiwan | Tertiary | Yeast isolates collected via Taiwan Surveillance of Antimicrobial Resistance of Yeasts (TSARY) | ND | 11 | 11/1139 (1%) among yeasts | ND |
| Zeng^83^ | 2019 | Retrospective cohort study | Single centre | 01/2013-12/2017 | China | Tertiary | Patients with invasive candidiasis | 243 | 12 | 12/243 (4.9%) among *Candida* spp. | ND |

^1,2,3,4^ Proportions (%) of *P. kudriavzevii* candidaemia over time were estimated from Figure 1 of the article^36^, Figure 1 of the article^31^, Supplementary Figure 2 of the article^13^, and Figure 1 of the article^35^, respectively.

Abbreviations: BTC= breakthrough candidaemia, ICU=intensive care unit, IFI=invasive fungal infection, NAC= non-*albican* candidaemia, ND=no data, TSARY= Taiwan Surveillance of Antimicrobial Resistance of Yeasts.
