# Supplementary material for: Pichia kudriavzevii (Candida krusei): A systematic review to inform the World Health Organisation priority list of fungal pathogens
Source: Med Mycol. 2024 Jun 27;62(6):myad132. doi: 10.1093/mmy/myad132 (PMC11210618; doi:10.1093/mmy/myad132)
Supplement: myad132_Supplemental_Files [file myad132_supplemental_files.zip › mm-2023-0240-File002.docx]

**Table 1. Risk of bias of included studies.**

| **Author** | **Year** | **Risk Level** |
| --- | --- | --- |
| Arendrup^27^ | 2013 | Low |
| Arikan-Akdagli^75^ | 2019 | Unclear |
| Awad^76^ | 2018 | Unclear |
| Badiee^19^ | 2017 | Unclear |
| Bassetti^11^ | 2011 | Low |
| Castanheira^28^ | 2020 | Low |
| Castanheira^29^ | 2014 | Low |
| Castanheira^24^ | 2014 | Unclear |
| Chen^77^ | 2017 | Unclear |
| Desnos-Ollivier^78^ | 2019 | Unclear |
| Fuller^36^ | 2019 | Low |
| Seyoum^26^ | 2020 | Unclear |
| Hrabovsky^79^ | 2017 | Low |
| Israel^21^ | 2019 | Low |
| Jung^30^ | 2020 | Low |
| Kakeya^31^ | 2018 | Unclear |
| Kaur^12^ | 2020 | Low |
| Kaur^23^ | 2020 | Unclear |
| Kronen^13^ | 2018 | Low |
| Lausch^32^ | 2018 | Low |
| Omrani^14^ | 2014 | Unclear |
| Orasch^33^ | 2018 | Low |
| Pfaller^80^ | 2011 | Unclear |
| Pfaller^25^ | 2015 | Unclear |
| Puig-Asensio^34^ | 2014 | Low |
| Salse^81^ | 2019 | Unclear |
| Sasso^37^ | 2017 | Low |
| Siopi^35^ | 2020 | Low |
| Tóth^22^ | 2019 | Unclear |
| van Schalkwyk^15^ | 2018 | Low |
| Yacoub^16^ | 2016 | Low |
| Yang^82^ | 2018 | Unclear |
| Zeng^83^ | 2019 | Low |

Table 2. Mortality associated with *P. kudriavzevii.*

| **Author** | **Year** | **Study design** | | **Study period** | **Country** | **Level of care** | **Population description** | **Number of patients** | **Number of** ***P. kudriavzevii* infected patients** | **Mortality (type, n/n, %)** |
| --- | --- | --- | --- | --- | --- | --- | --- | --- | --- | --- |
| Bassetti^11^ | 2011 | Prospective cohort study | Single centre | 01/2008-12/2010 | Italy | Tertiary | Patients with candidaemia | 348 | 9 | 5/9 (55.5%) |
| Kaur^12^ | 2020 | Retrospective cohort study | Single centre | 01/2014-12/2014 | India | Tertiary | Adult and paediatric patients with candidaemia | 316 (n=186 paediatric, 130 adults) | 316 | Paediatric patients:  17/74 (22.94%)  Adult patients: not reported |
| Kronen^13^ | 2018 | Retrospective cohort study | Single centre | 01/2002-01/2015 | United States | Tertiary | Patients with candidaemia | 1873 | 59 | 90-day all-cause mortality for bloodstream infection (BSI): 64.40% |
| Omrani^14^ | 2014 | Retrospective cohort study | Single centre | 01/2003-12/2012 | Saudi Arabia | Tertiary | Patients with invasive *Candida* infections | 652 | 9 | 30-day mortality: 4/9 (44%) 90-day mortality: 6/9 (67%) |
| van Schalkwyk^15^ | 2018 | Retrospective cohort study | Single centre | 01/2012-12/2016 | South Africa | Tertiary | Neonates with bloodstream infections during multiple outbreaks | 589 during the first outbreak | 48 | 7/48 (14.6%) |
| Yacoub^16^ | 2016 | Retrospective cohort study | Single centre | 01/2001-06/2014 | United States | Tertiary | Cancer patients with candidaemia | 247 | 32 | 19.23% |

Table 3. Studies reporting drug susceptibility of *P. kudriavzevii.*

| **Author** | **Year** | **Study design** | | **Study period** | **Country** | **Level of care** | **Population description** | **Number of patients** | **Number of isolates** | **Number of** ***P. kudriavzevii* isolates** | **Samples collected from** |
| --- | --- | --- | --- | --- | --- | --- | --- | --- | --- | --- | --- |
| Arendrup^27^ | 2013 | Prospective national surveillance study | Multi-centre | 2010-2011 | Denmark | Tertiary | Patients with fungaemia | 995 | 1081 fungal isolates | 52 | Blood |
| Arikan-Akdagli^75^ | 2019 | Retrospective cohort study | Multi-centre | 1997-2017 | Turkey | Tertiary | *Candida* spp. isolates from 12 centres | ND | 1991 *Candida* spp. | 52 | Blood |
| Badiee^19^ | 2017 | Cross sectional study | Multi-centre | 2014-2015 | Iran | Tertiary | Immunocompromised patients admitted to 10 hospitals in Iran | ND | 846 *Candida* spp. | 23 | Various sites (blood, CSF, bronchoalveolar lavage, and sputum) |
| Castanheira^28^ | 2020 | Global surveillance study | Multi-centre | 01/2016-12/2017 | Asia Pacific, Europe, Latin America, North America | Tertiary | Patients with *Candida* infections (from 60 hospitals in 25 countries) | 2936 | 2936 *Candida* spp. | 76 | Various sites (majority blood) |
| Castanheira^29^ | 2014 | Global surveillance study | Multi-centre | 2012 | Europe, Latin America, North America and the Asia-Pacific Region | Tertiary | Patients with invasive fungal infections | ND | 1717 | 36 | Various (blood, sterile body fluids, tissues, abscesses, respiratory tract) |
| Castanheira^24^ | 2014 | Cross sectional study | Multi-centre | 2012 | North America, Europe, Latin America, and the Asia Pacific region | Tertiary | Patients with *Candida* spp. infection (from 75 medical centres globally) | ND | 1421 | 32 | Various (blood, sterile body fluids, tissues, abscesses) |
| Chen^77^ | 2017 | Retrospective cohort study | Single centre | 01/2007-12/2012 | Taiwan | Tertiary | Patients with candidaemia | ND | 709 *Candida* spp. | 13 | Blood |
| Desnos-Ollivier^78^ | 2019 | Retrospective cohort study | Multi-centre | 01/2015-10/2017 | France | Tertiary | Patients with invasive infections | ND | 1457 | 76 | Blood (majority), CSF and other |
| Fuller^36^ | 2019 | Prospective cohort study | Multi-centre | 01/2011-10/2016 | Canada | Tertiary | Patients with bloodstream infections | ND | 1882 *Candida* spp. | 81 | Blood |
| Seyoum^26^ | 2020 | Retrospective cohort study | Multi-centre | 01/2018-09/2018 | Ethiopia |  | Patients with yeast isolated | ND | 209 yeast | 14 |  |
| Hrabovsky^79^ | 2017 | Retrospective cohort study | Single centre | 01/2013-06/2015 | Slovakia | Tertiary | Adult non-neutropenic ICU patients | 426 | 800 yeasts | 69 | Sterile (n=101), non-sterile body sites (n=699) |
| Israel^21^ | 2019 | Retrospective cohort study | Multi-centre | 01/2005-12/2016 | Israel | Tertiary and secondary | Patients with candidaemia | 899 | 919 *Candida* spp. | 54 | Blood |
| Kaur^12^ | 2020 | Retrospective cohort study | Single centre | 01/2014-12/2014 | India | Tertiary | Adult and paediatric patients with candidaemia | 316 (n=186 paediatric, 130 adults) | 316 *Candida* spp. | 96 | Blood |
| Kaur^23^ | 2020 | Retrospective cohort study | Single centre | 01/1999-12/2018 | India | Tertiary | Patients with candidaemia | 7927 | 7927 | 527 | Blood |
| Omrani^14^ | 2014 | Retrospective cohort study | Single centre | 01/2003-12/2012 | Saudi Arabia | Tertiary | Patients with invasive *Candida* infections | 652 | 800 *Candida* spp. | 9 | Sterile sites (blood, CSF, other body fluid, tissue biopsies) |
| Pfaller^80^ | 2011 | Retrospective cohort study | Multi-centre | 01/2008-12/2009 | Asia-Pacific (16 centres, 51 isolates), European (25 centres, 750 isolates), Latin American (10 centres, 348 isolates) and North American (28 centres, 936 isolates) regions. | Tertiary | Patients with candidaemia reported under global surveillance | 1752 | 1752 | 36 | Blood |
| Pfaller^25^ | 2015 | Retrospective cohort study | Multi-centre | 2013 | North America (695 isolates, 29 sites), Europe (511 isolates, 19 sites), the Asia-Pacific region (222 isolates, 12 sites), and Latin America (185 isolates, 10 sites). | Tertiary | Patients with invasive fungal infections | 1320 | 1320 *Candida* spp. | 37 | Blood (majority), sterile body fluids (CSF, pleural and peritoneal fluids), tissues, abscesses, respiratory tract and other |
| Salse^81^ | 2019 | Retrospective cohort study | Multi-centre | 2004-2018 | France | Tertiary | Patients with infections by yeast and *Aspergillus fumigatus* species from 12 French hospitals | ND | 575 | 575 | Blood, sterile sites and other sites, such as bronchoalveolar lavage, sputum |
| Sasso^37^ | 2017 | Retrospective cohort study | Single centre | 2007-2016 | France | Tertiary | ICU patients with invasive *Candida* infections | 244 | 3557 | 192 | Blood, other sterile sites |
| Tóth^22^ | 2019 | Retrospective cohort study | Single centre | 01/2005-12/2018 | Hungary | Tertiary | Patients with *P. kudriavzevii* isolates collected | 53 | 53 | 53 | Sterile body sites (blood, cerebrospinal, pleural and peritoneal fluids, deep wounds, etc.) |

Abbreviations: CSF=cerebrospinal fluid, ND=no data, ICU=intensive care unit.

Table 4. Drug susceptibility of *P. kudriavzevii* to azoles.

| **Author** | **Year** | **MIC method** | **Fluconazole** | **Isavuconazole** | **Itraconazole** | **Posaconazole** | **Voriconazole** |
| --- | --- | --- | --- | --- | --- | --- | --- |
| Arendrup^27^ | 2013 | EUCAST (EUCAST BP, CLSI BP for itraconazole) | (n=52) 0% S | ND | (n=52) 28.8% S | (n=52) 3.8% S | (n=52) 11.5% S |
| Arikan-Akdagli^75^ | 2019 | CLSI | (n=52) GM MIC (range): 27.64 (8- >64), 100% R | ND | 0.17 (≤0.015-0.5), 0% non-WT | 0.14 (≤0.03-1), 1.9% non-WT | 0.07 (0.03-0.125), 100% S |
| Badiee^19^ | 2017 | CLSI (susceptibility based on CLSI BP, or ECV) | GM MIC (range):  17.9 (2-64), >64 (5%) non-WT | ND | 0.2 (0.064-1), 33.3% R | 0.126 (0.032-0.5), >0.5 (5%) non-WT | 0.284 (0.032-16), 20% R |
| Castanheira^28^ | 2020 | CLSI | ND | ND | ND | 0% non-WT | 1.3% R (5% R in North America, n=20) |
| Castanheira^29^ | 2014 | CLSI | ND | ND | ND | 5.6% R | 2.8% R |
| Castanheira^24^ | 2014 | CLSI | ND | MIC/MEC range: 0.12-2, MIC/MEC_50_: 0.5, MIC/MEC_90_: 0.5, % not available | 0.25-4, 0.25, 0.5, 3.1% non-WT | 0.12-2, 0.25, 0.5, 6.3% non-WT | 0.12-4, 0.25, 0.25, 3.1% non-WT |
| Chen^77^ | 2017 | Sensititre YeastOne | MIC range: 32-128, MIC_50_: 64, MIC_90_: 64 Considered intrinsically resistant | ND | ND | ND | 0.12-0.5, 0.5, 0.5, 0% R 100% S |
| Desnos-Ollivier^78^ | 2019 | EUCAST | MIC range:16- ≥64, MIC_50_: 32, MIC_90_: 64 %R not available (considered intrinsically resistant) | MIC range:0.015-1, MIC_50_: 0.125, MIC_90_: 0.25 %isolates with MIC>MIC_90_: 6.58% | ND | ND | ND |
| Fuller^36^ | 2019 | CLSI | mode MIC: 8, MIC_90_: 16 | ND | ND | ND | MIC_90_: 0.25 |
| Seyoum^26^ | 2020 | VITEK 2 compact system | n=14, 100% R | ND | ND | ND | 0% R |
| Hrabovsky^79^ | 2017 | EUCAST | (n=40 isolates for invasive disease) MIC range: 2-256, MIC_50_: 256, MIC_90_: 256 100% R | ND | ND | ND | 0.094-4, 0.5, 1, 5% R |
| Israel^21^ | 2019 | CLSI | NA (considered intrinsically resistant) | ND | ND | ND | (n=54), 3.8% R |
| Kaur^12^ | 2020 | CLSI | ND | ND | (n=82 paediatric isolates)  GM MIC (range): 0.31 (0.12-0.5), MIC_50_: 0.25, MIC_90_: 0.5 | 0.24 (0.06-0.5), 0.25, 0.5 | 0.41 (0.05-8), 0.25, 0.25 |
| Kaur^23^ | 2020 | CLSI | For 2014-2018 period: 40.5% R | ND | 4.2% R | 0% R | 1.9% R |
| Omrani^14^ | 2014 | CLSI | n=13, 0% S | ND | ND | ND | n=6, 100% S |
| Pfaller^80^ | 2011 | CLSI | ND | ND | ND | n=16 ICU, 0% R  n=20 non-ICU, 0% R | n=16 ICU, 0% R  n=20 non-ICU, 0% R |
| Pfaller^25^ | 2015 | CLSI | MIC/MEC range: 8- >128, MIC_50_: 32, MIC_90_: 64, Intrinsically resistant. | MIC/MEC range: 0.12-4, MIC_50_: 0.5, MIC_90_: 1 | MIC/MEC range: 0.25-2, MIC_50_: 0.5, MIC_90_: 1, 2.7% non-WT 97.3% WT | MIC/MEC range: 0.25-1, MIC_50_: 0.5, MIC_90_: 0.5, 2.7% non-WT, 97.3% WT | 2.7%R, 94.6% S |
| Salse^81^ | 2019 | E-test | n=414, mode MIC: >256 | ND | ND | ND | n=575, mode MIC: 0.5 |
| Sasso^37^ | 2017 | E-test (CLSI BP) | 100% R (n=48) (averaged for 2007-2016) | ND | ND | ND | 79.4% S (n=55) 29.6% I (n=47) (averaged for 2007-2016) |
| Tóth^22^ | 2019 | CLSI | mode MIC (range): 32 (8- >32), MIC_50_: 32 MIC_90_: >32 %R ND | ND | ND | ND | ND |

Data are reported as they appear in source documents. Susceptibility is expressed as mg/L unless indicated otherwise. Abbreviations: BP=breakpoint, CLSI=Clinical and Laboratory Standards Institute, ECV=epidemiological cutoff value, EUCAST= European Committee on Antimicrobial Susceptibility Testing, R=resistant, S=susceptible, S-DD=susceptible dose-dependent, I=intermediate, MIC= minimum inhibitory concentration, MEC=minimum effective concentration, GM= geometric mean, NA/ND= not applicable / not done, MIC_50_=MIC required to inhibit the growth of 50% of isolates, MIC_90_=MIC required to inhibit the growth of 90% of isolates, ND= no data, Non-WT = non wild-type.

Table 5. Drug susceptibility of *P. kudriavzevii* to non-azole antifungal drugs.

| **Author** | **Year** | **MIC method** | **Anidulafungin** | **Caspofungin** | **Micafungin** | **Amphotericin B** | **Flucytosine** |
| --- | --- | --- | --- | --- | --- | --- | --- |
| Arendrup^27^ | 2013 | EUCAST (EUCAST BP, CLSI BP for caspofungin and itraconazole) | (n=52) 100% S | (n=25) 28% S | ND | (n=52) 73.1% S | ND |
| Arikan-Akdagli^75^ | 2019 | CLSI | ND | ND | 0.08 (≤0.03-0.25), 100% S | 1.32 (0.5-2), 0% non-WT | ND |
| Badiee^19^ | 2017 | CLSI (susceptibility based on CLSI BP, or ECV) | ND | 0.2 (0.032-2), 30% R | ND | 1.004 (0.032-8), 40% R | ND |
| Castanheira^28^ | 2020 | CLSI | 0% R | 0% R | 0% R | 0% non-WT | ND |
| Castanheira^29^ | 2014 | CLSI | 2.8% R | 2.8% R | 0% R | ND | ND |
| Castanheira^24^ | 2014 | CLSI | 0.03-1, 0.06, 0.12, 3.1% non-WT | 0.06-1, 0.12, 0.25, 3.1% non-WT | 0.015-0.12, 0.12, 0.12, 0% non-WT | 1-2, 1, 2, 0% non-WT | 8-32, 16, 16, 0% non-WT |
| Chen^77^ | 2017 | Sensititre YeastOne | 0.12-0.25, 0.12, 0.12, 0% R 100% S | 0.25-0.5, 0.5, 0.5, 0% R 23.1%S 76.9% I | 0.6-0.12, 0.12, 0.12, 0% R 100% S | ND | ND |
| Fuller^36^ | 2019 | CLSI | ND | MIC not available, 0% R | MIC not available, 0% R | MIC not available, 100% WT (based on ECV ≤2) | ND |
| Seyoum^26^ | 2020 | VITEK 2 compact system | ND | 0% R | 0% R | ND | 78.6% R |
| Hrabovsky^79^ | 2017 | EUCAST | 0.002-0.19, 0.008, 0.023, 5% R | 0.002-0.25, 0.063, 0.125, %R ND | ND | 0.19-2, 0.5, 1, 5% R | ND |
| Israel^21^ | 2019 | E-test (CLSI BP) | ND | 67% R | ND | 1.9% R | ND |
| Kaur^12^ | 2020 | CLSI | 0.28 (0.03-4), 0.12, 0.5 | 0.35 (0.12-2), 0.12, 0.5 | 0.45 (0.06-12), 0.12, 0.5 | 0.90 (0.25-2), 1, 1 | ND |
| Kaur^23^ | 2020 | CLSI | 1.9% R | 16% R | 2.5% R | 12.9% R | ND |
| Omrani^14^ | 2014 | CLSI | ND | n=6, 66.7% S | ND | n=14, 100% S | ND |
| Pfaller^80^ | 2011 | CLSI | n=16 ICU, 0% R  n=20 non-ICU, 0% R | n=16 ICU, 6.3% R  n=20 non-ICU, 5.0% R | n=16 ICU, 0% R  n=20 non-ICU, 0% R | ND | ND |
| Pfaller^25^ | 2015 | CLSI | 0% R, 100% S | 0% R, 100% S | 0% R, 100% S | MIC/MEC range: 1-2, MIC_50_: 1, MIC_90_: 2, 0% non-WT, 100% WT | MIC/MEC range: 8-32, MIC_50_: 16, MIC_90_: 32, 0% non-WT, 100% WT |
| Salse^81^ | 2019 | E-test | n=117, mode MIC: 0.03 | n=565, mode MIC: 0.5 | n=259, mode MIC: 0.25 | n=534, mode MIC: 1 | ND |
| Sasso^37^ | 2017 | E-test (CLSI BP) | ND | 62.6% S (n=31) 86.8% I (n=50) (averaged for 2007-2016) | ND | 100% WT (n=51) | ND |
| Tóth^22^ | 2019 | CLSI | 0.06 (0.015-0.25), 0.06, 0.12, 100% S | 1 (0.12-1), 1, 1, 11.3% S 22.6% I 66.1% R | 0.25, (0.03-0.25), 0.25, 0.25,  100% S | 1 (0.5-2), 1, 1 | ND |

Data are reported as they appear in source documents. Susceptibility is expressed as mg/L unless indicated otherwise.

Abbreviations: BP=breakpoint, CLSI= Clinical and Laboratory Standards Institute, ECV=epidemiological cutoff value, EUCAST= European Committee on Antimicrobial Susceptibility Testing, R=resistant, S=susceptible, S-DD=susceptible dose-dependent, I=intermediate, ICU=intensive care unit, MIC= minimum inhibitory concentration, MEC=minimum effective concentration, GM= geometric mean, NA/ND= not applicable / not done, MIC_50_=MIC required to inhibit the growth of 50% of isolates, MIC_90_=MIC required to inhibit the growth of 90% of isolates, ND= no data, Non-WT = non wild-type.

**Table 6. Risk factors for infections caused by *P. kudriavzevii.***

| **Author** | **Year** | **Study design** | | **Study period** | **Country** | **Level of care** | **Population description** | **Number of patients** | **Number of *P. kudriavzevii* isolates** | **Risk factors** |
| --- | --- | --- | --- | --- | --- | --- | --- | --- | --- | --- |
| Kaur^12^ | 2020 | Retrospective cohort study | Single centre | 01/2014-12/2014 | India | Tertiary | Adult and paediatric patients with candidaemia | 316 (n=186 paediatric, 130 adults) | 316 | Significantly greater prevalence in paediatric group (44%, 82/186) vs adults (10.8%, 14/130; p<0.001).  Gastrointestinal disease (p = 0.018),  Prior use of antibiotics (p = 0.021),  Exposure to carbapenems (p = 0.039). |
| Kaur^23^ | 2020 | Retrospective cohort study | Single centre | 01/1999-12/2018 | India | Tertiary | Patients with candidaemia | 7927 | 527 | Paediatric patients:  422/527 (80.1%) paediatric vs 105/527 (19.9%) adults |
| Kronen^13^ | 2018 | Retrospective cohort study | Single centre | 01/2002-01/2015 | US | Tertiary | Patients with candidaemia | 1873 | 59 | Six variables (multivariate analysis):  Hematologic malignancy (OR, 10.7; 95% CI, 5.1-22.4),  gastric malignancy (OR, 14.7; 95% CI, 3.0-72.8),  neutropenia (OR, 2.1; 95% CI, 1.1-4.1),  prior azole use (OR, 2.4; 95% CI, 1.2-4.7),  prior monoclonal antibody use (OR, 5.4; 95% CI, 2.0-14.9), and β-lactam/β-lactamase inhibitor use (OR, 2.4; 95% CI, 1.3-4.7) within 90 days prior to *Candida* BSI. |
| Lausch^32^ | 2018 | Retrospective cohort study | Multi-centre | 2010-2011 | Denmark | Mixed (data from national surveillance) | Adult patients with candidaemia | 841 | 35 | Prior antifungal treatment (AFT):  Substantially higher in patients with prior AFT ([12.9% for azoles and 9.1% for echinocandins] vs 2.2% without prior AFT) |
| van Schalkwyk^15^ | 2018 | Retrospective cohort study | Single centre | 01/2012-12/2016 | South Africa | Tertiary | Neonates with blood-stream infections during multiple outbreaks | 589 during the first outbreak | 48 | With *P. kudriavzevii* candidaemia vs Without:  Necrotizing enterocolitis (aOR 3.1, 95%CI 1.4-6.7),  Birthweight (in reference to >2.5kg):  extreme low <1kg (aOR 6.5, 95%CI 1.9-21.6),  1- <1.5 kg (6.1 (2.1-17.2)),  1.5-1.9 kg (3.4 (1.1-10.0)) |

Abbreviations: AFT=antifungal treatment, aOR=adjusted odds ratio, OR=odds ratio, BSI=bloodstream infection.
